# Knowledge, Attitudes, and Experiences of HIV Pre-Exposure Prophylaxis (PrEP) Trial Participants in Botswana

**DOI:** 10.4236/wja.2015.51002

**Published:** 2015-02-12

**Authors:** Lauren Toledo, Eleanor McLellan-Lemal, Faith L. Henderson, Poloko M. Kebaabetswe

**Affiliations:** 1 ICF International, Atlanta, USA; 2 Division of HIV/AIDS Prevention, National Center for HIV, Viral Hepatitis, STD, and TB Prevention, Centers for Disease Control and Prevention (CDC), Atlanta, USA; 3 HIV Prevention Research Unit, CDC Botswana, Gaborone, Botswana; 4 Faculty of Medicine, University of Botswana, Gaborone, Botswana

**Keywords:** Pre-Exposure Prophylaxis, HIV/AIDS, Botswana, Qualitative Research

## Abstract

Recent clinical trials have shown that a daily dose of oral TDF/FTC pre-exposure prophylaxis (PrEP) is effective in reducing human immunodeficiency (HIV) risk. Understanding trial participants’ perspectives about retention and PrEP adherence is critical to inform future PrEP trials and the scale-up and implementation of PrEP programs. We analyzed 53 in-depth interviews conducted in April 2010 with participants in the TDF2 study, a Phase 3, randomized, double-blind, placebo-controlled clinical trial of daily oral TDF/FTC with heterosexual men and women in Francistown and Gaborone, Botswana. We examined participants’ knowledge, attitudes, and experiences of the trial, identified facilitators and barriers to enrollment and retention, and compared participant responses by study site, sex, and study drug adherence. Our findings point to several factors to consider for participant retention and adherence in PrEP trials and programs, including conducting pre-enrollment education and myth reduction counseling, providing accurate estimates of participant obligations and side effect symptoms, ensuring participant understanding of the effects of non-adherence, gauging personal commitment and interest in study outcomes, and developing a strong external social support network for participants.

## 1. Introduction

Recent clinical trials have shown that a daily dose of oral TDF/FTC (Truvada^®^) pre-exposure prophylaxis (PrEP) is effective in reducing human immunodeficiency virus (HIV) infection risk among men who have sex with men (MSM), heterosexual men and women, and injecting drug users [1]-[4]. Two additional clinical trials conducted among African women failed to show efficacy [5] [6]. Results from these trials suggest that there is a strong dose response between PrEP use and HIV protection [7], and that PrEP is an effective method for HIV prevention when adherence is high [8]. Efforts are currently underway to scale-up PrEP programs [7].

Understanding participants’ perspectives about adherence to PrEP and compliance with clinical trial visits is critical, not only to ensure rigorous scientific method and confidence in clinical trial findings, but also to inform the implementation and scale-up of PrEP programs. In the TDF2 Trial, a Phase 3 clinical trial of daily oral TDF/FTC with sexually active heterosexual men and women in Botswana, the efficacy of TDF/FTC in a modified intention-to-treat analysis was 62.2% [3]. The TDF2 trial had experienced lower than expected rates of retention, which led to an earlier than planned study closure and questions about participants’ willingness to participate and retention in future PrEP implementation efforts in Botswana [3]. Current PrEP Guidelines in the United States recommend that patients taking PrEP engage regularly with health care providers, including HIV testing every 3 months, renal function assessment at baseline and every six months, and access to risk-reduction services [9]. Efforts to understand the social and environmental contexts of clinical trial participant experiences and perspectives may help to identify barriers and facilitators to correct PrEP use in the “real world”.

In-depth interviews and focus-group discussions with participants in HIV PrEP clinical trials with MSM in Thailand and San Francisco identified factors affecting PrEP adherence, including motivators for study participation, knowledge of the study product and study objectives, medication concerns, participant schedules, and social stigma and support [10] [11]. Participants in clinical trials of microbicides for the prevention of HIV infection have identified age, education, waiting periods at the clinics, rumors, others’ opinions of the study, relationship with study staff, confirmation of good health, and access to health education as factors that influence attendance and retention [12]–[14].

Identifying social, behavioral, and environmental factors related to adherence and retention of participants in PrEP trials will inform strategies for adherence and retention counseling for future PrEP studies as well as for the roll out of any “real world” PrEP programs in Botswana. This paper describes TDF2 participants’ knowledge, attitudes, and experiences of the TDF2 trial and examines how these perspectives may relate to trial retention and study drug adherence. We identified facilitators and barriers to enrollment and retention and compared participant responses by study site, sex, and study drug adherence.

## 2. Methods

### 2.1. Design

The TDF2 Study was a Phase 3, randomized, double-blind, placebo-controlled clinical trial of daily oral TDF/FTC with 1219 sexually active heterosexual men and women in Francistown and Gaborone, Botswana that took place between February 2007 and May 2010. Eligibility criteria, the recruitment process, and study protocols are described in detail elsewhere [3]. We conducted in-depth interviews in April 2010 in Gaborone and Francistown for the qualitative analysis described in this report.

This paper focuses on attitudes toward and experiences of participants enrolled in the TDF2 trial. Specifically, our analysis examined open-ended responses to two domains of inquiry: 1) pre-enrollment attitudes and knowledge about the trial purpose and 2) facilitators and barriers to taking part in and remaining in the study. Interview guide questions included in the analysis are provided in Table 1. We have included trial data to describe the sample demographically and to classify participants’ self-report of non-adherence to the study drug. In the interview guide and throughout this paper, references to pills, study pills, and study drug include both Truvada^®^ and placebo.

### 2.2. Participants and Data Collection

We selected participants for the in-depth interviews using a simple random sampling. To be eligible for consideration for an in-depth interview, participants must have completed at least six months of trial participation and had attended all of their study visits. To optimize the range of trial participants’ experiences, we used two behavioral criteria (sexual behavior change and adherence to study drug) to first classify participants. Sexual behavior change was assessed on three variables from the quantitative trial data: number of sexual partners, times condoms were used with a main partner and times condoms were used a casual partner. Participants with a percentage increase in one or more of these behaviors between baseline data collection and the month 3 visit were classified as *high risk*; those with no change or a percentage decrease were classified as *neutral/low risk*. For adherence to study drug, participants who self-reported missing any doses or sharing study drugs over a three-month period (baseline to month 3 visit or month 3 visit to month 6 visit) were classified as *low adherers*; those who self-reported neither missing any doses nor sharing study drugs were considered *adherers*. Participant IDs were stratified by gender and entered into separate two-by-two contingency tables for each study site using SAS version 9.2 (SAS Institute, Cary, North Carolina, USA). Three participant IDs were randomly selected from each cell. From the unselected participant IDs, we then randomly selected with no consideration to our behavioral classification scheme three male and three female participant IDs. No participants declined participation in an in-depth qualitative interview. A total of 30 potential interview participants (equally distributed by gender) were identified for each site. Study staff recruited these participants for the qualitative sub-study during a monthly study visit before they exited the study.

The Botswana Health Research and Development Committee and an institutional review board at the US Centers for Disease Control and Prevention reviewed and approved the protocol, consent forms, and supporting documents. All in-depth interview participants provided written informed consent prior to their interview. We conducted interviews in Setswana or English, at the participants’ discretion. Interviews were audio recorded and took place in the trial clinic and lasted between 45 and 60 minutes. We provided participants food and drink during their interview and 5 Pula (0.72 USD) after completing the interview.

### 2.3. Data Analysis

Interviewers transcribed the audio-recorded interviews verbatim in the original data collection language and then translated the transcription into English for analysis if needed. An experienced qualitative analyst performed an inductive thematic analysis [15] in NVivo 8 using a four-step approach. A single analyst was used, given budgetary considerations. The initial step focused on developing and applying structural codes to the English transcripts. Structural codes identified the question and corresponding response for each interview guide item, and provided contextual information for the second coding step, which involved an iterative constant comparison approach [16]. Before undertaking the content-based coding, the analyst reviewed all transcripts in detail from beginning to end and prepared margin notes to identify both unique and recurrent concepts. She then used concepts identified through this process to develop an emergent, data-driven codebook to guide the application of the content-related codes. The analyst reviewed and revised both code definitions and code applications throughout the analysis process to ensure that the coding scheme adequately fit the data. The third step, data interpretation, involved identifying salient and co-occurring concepts and relationships between concepts, which were then categorized into themes. In the final step, the analyst compared thematic findings by study site (Francis-town and Gaborone), sex (male and female), and self-reported adherence data from the Adult AIDS Clinical Trials Group (AACTG) Adherence Questionnaire [17], which researchers collected at each monthly visit through a face-to-face interview for the 36-month trial duration. Findings for these quantitative data have been published elsewhere [18]. Information presented here is only for the qualitative sub-study participants.

## 3. Results

We analyzed 53 interviews: Twenty-six (49%) from Francistown and 27 (51%) from Gaborone. As shown in Table 2, 77% (n = 41) of the interviewees were in the 21 – 29 age group and 94% (n = 50) had attended secondary or post-secondary school. More than 60% had participated in the clinical trial for at least 13 months at the time they were interviewed. During one or more monthly study visits, 36 out of the 53 (68%) in-depth interview participants reported non-adherence (*i.e.*, missed one or more doses) to the study drug.

We present the themes from the thematic analysis below, organized by the two domains of inquiry: Pre-enrollment knowledge and attitudes about the trial and facilitators and barriers to trial participation. Differences in the emerging thematic findings by study site, sex, and adherence are noted when present.

### 3.1. Pre-Enrollment Knowledge and Attitudes about Trial

Participants said they learned of the TDF2 trial from TV and radio advertisements, friends and neighbors, and from trial recruitment staff. Gaborone participants said that these sources provided information about the trial’s aims and participant eligibility requirements (e.g., age, HIV status). One woman shared, “I only knew that only HIV-negative people can join and that they wanted to try a pill to find out if it can work to prevent HIV/AIDS and that this pill (Truvada^®^) is taken by… some people who are suffering from AIDS.” Many participants, however, explained that although they currently felt well informed about the study, they did not have an in-depth understanding until they made their first visit to the study clinic. Female participants, in particular, mentioned that they did not have “any details” about the trial. Some Francistown participants shared that they received misinformation from peers about the study that was corrected upon their first visit to the study clinic. A woman from Francistown said, “[I did not have] valuable information [prior to joining the study]. It was mostly assumptions about how things are and when you get [to the clinic] you find out that things are not the same as how you thought. I thought we would be given ARVs [treatment for HIV], and then you think you will be given ARVs when you do not have the virus, [which would cause] a problem.”

When asked about beliefs circulating in the community about the trial, participants focused on the negative perceptions held toward trial researchers. These perceptions centered on the belief that American TDF2 researchers were using members of the community as “experimental test subjects” and were exposing trial participants to HIV, either through injections, study pills containing HIV, or high-risk sexual contact (e.g., having sex without a condom or with an HIV-infected partner). In addition, participants explained that some viewed the TDF2 trial as evidence that researchers believed that community members were gullible and would enroll in any type of research study regardless of the risks. A female from Gaborone explained, “They have a tendency of saying that Batswana [Bantu people living in Botswana] are stupid, that the white man is scared of testing the virus on themselves and prefer to try it on us.” Another belief was that the trial was an attempt by foreign governments to reduce the population. Although these negative perceptions of trial researchers were discussed by all participants, they were mentioned more often by Gaborone participants. Less than a quarter of Francistown participants said they were aware of any negative community views of the trial.

### 3.2. Facilitators and Barriers to Trial Participation

We classified emerging themes in participant discussions of facilitators and barriers to trial participation into three categories: 1) enrollment concerns, 2) facilitators to enrollment and retention, and 3) challenges experienced during participation.

Participants discussed three main concerns about enrolling in the trial: 1) discouragement from others, 2) side effects, and 3) fear of disease infection. Across all interviews, participants described negative reactions or discouragement from friends and family regarding involvement with the study. Participants attributed increased apprehensions about taking part in the trial to this discouragement. Participants explained that negative reactions by others were often related to the aforementioned suspicion about the intentions of trial researchers, in particular the risk of being deliberately exposed to the virus. A male from Gaborone shared, “The people that I live with, they criticize the study by saying that ‘those people will infect you with the virus,’ things like that… If someone does not see things the way I see them, they can derail that person [from participating]…”

Some participants expressed general concerns about the potential short- and long-term side effects of taking the study drug. Participants did not focus on specific side effects, but were concerned that the pills could weaken their body or negatively affect them in some way. A female from Francistown explained, “The disadvantage [of participation] is that this pill, nobody knows whether is going to work or not… They might have those side effects… those side effects might affect my life for good.” Non-adherent participants in the qualitative sample were less likely than others to mention side effect concerns.

Related to community mistrust of trial researchers’ intentions, participants’ infection apprehensions focused on the study drug or trial procedures. Participants, in particular those who were non-adherent to the study drug, indicated that they were afraid that exposure to HIV or another non-specified fatal disease would result. A female from Gaborone said, “I thought participants would be injected with positive blood.” A male from Gaborone said, “I suspected that Truvada was somehow injected with HIV.” Participants in Gaborone also questioned the ability of a clinical trial to test the efficacy of the study drug on HIV-uninfected participants without exposing them to HIV. A female stated, “It’s just that I don’t know where we are going to find [the pill’s] benefit because we are not allowed to have sex with people who have the virus. If we were allowed to have sex with people who have HIV, perhaps we could say it was effective, we could tell that the pill could prevent [HIV].” Nearly all participants who expressed concerns about HIV infection also described having friends or family discourage their involvement in the study based on fears about HIV exposure.

### 3.3. Facilitators to Enrollment and Retention

The majority of participants at both study sites characterized their involvement with the TDF2 trial as a positive experience. These participants described positive interactions with study staff, as noted by this female participant from Gaborone, “Since I started coming to the clinic, I have never been offended by anything or by anyone who was helping me. The people who were helping me had a loving spirit… even when I asked questions they were answering me with love… They really had good relations. They did not force anything.” In addition to positive interactions with trial staff, participant discussions about facilitators or motivators to take part and/or remain in the TDF2 trial centered on five factors: 1) access to medical services, 2) altruism, 3) personal commitment, 4) efficacy interest, and 5) support and encouragement.

All participants with the exception of one identified the opportunity to access health services as a reason for seeking enrollment in the trial. Specifically, participants believed that the trial would provide access to regular HIV testing, general health checkups, health counseling, and protection against HIV. A female from Gaborone said, “What encouraged me is that when we get [to the study clinic] we are tested for diseases and pregnancy. You get motivated that hidden diseases will be detected and treated.” In addition, some participants liked the opportunity to access a private trial clinic as opposed to a government-sponsored clinics or hospital where test results may take a long time. Some male participants were motivated to enroll because they had an HIV-infected sex partner and felt they were at risk for HIV infection. These men looked forward to the possibility that the trial could provide protection against HIV (either from the study pill or through the trial-supported risk-reduction counseling). A male from Gaborone explained, “The first reason that put me in a position to join the study is that my partner lives with HIV… I ended up joining the study because I wanted to save my life. [I joined] so that I could benefit from taking the pills or following health instructions.”

Participants noted that that their expectations with regard to access to medical services were met and provided motivation to stay enrolled in the trial. Many participants in Gaborone appreciated the trial staff’s commitment to keeping their health information confidential. One woman explained, “At the local clinics the employees have friends in the village and when you come as a patient they can gossip about you saying ‘that one is sick from this or that.’ At BOTUSA [study clinic] our secrets were only between the participant and the staff.”

Participants stated that they had a desire to volunteer and contribute to Botswana’s HIV prevention efforts. These participants focused on Botswana’s high HIV prevalence, like this male from Gaborone who said, “The virus is spreading on daily basis in our country. I thought that my participation would help a lot of young people… to reduce transmission of HIV.” Participants described short-term altruistic motives, or participating to help researchers test the efficacy of “the pill”, as well as long-term altruism, which focused on participation as an opportunity to contribute to the discovery of an effective pill that would ultimately reduce HIV infection in Botswana. As a woman from Francistown said, “If this pill works I will have helped the nation.”

A majority of participants attributed their ability to remain in the study to a personal commitment or internal decision to see it through. A female from Gaborone explained, “I had committed myself. I came in whole heartedly. When you do something not because someone told you to do it, it becomes very easy… I was really committed.” Another Gaborone female said, “It comes deeply from you whether you want. If you want you will continue visiting.”

Another motivator for enrolling or staying enrolled in the trial was interest in the efficacy of the study product. Participants indicated that they were curious about the study results or wanted “to find out whether this pill would really work or not.” A male from Gaborone shared, “I joined this study to see the benefit at the end. So that at the end I could see exactly what was being researched and if indeed it has been found out. This is what kept me in the study for this long.”

The majority of participants described receiving support or encouragement from someone in their life to enroll or continue participation in the trial. Participants explained that although some friends and family were initially suspicious about the aims of the trial, providing in-depth explanations about the research methods and study drug often resulted in acceptance and support for their involvement. A female from Gaborone told how her sexual partner reacted, “He is encouraging me. I mean he didn’t understand what I really meant by this study and what is done, so I told him, ‘In this study 1, 2, 3, 4 is being done. Taking this pill does not mean I have the virus. If you don’t believe that I don’t have the virus…you [can] go with me to the study.’ And he went with me. That’s when he understood.” A female from Francistown likewise explained, “[My family] encouraged me to stand up and test myself and I told them that there is a clinical trial that is said to have a pill that prevents the virus and they said I should go there.”

Many of those who self-reported adherence to the study pills described having friends and family reinforce messages received during study counseling sessions, such as encouraging safer sexual practices (e.g., reduce number of partners, use condoms). A female from Gaborone, who self-reported that she had been adherent to the study pills, shared, “They support me… my mother would peep in and say, ‘Who are you walking with today, haven’t they said you should reduce [your sexual partners]?’ My mother is an open person; she would peep in and say, ‘Hey girl, have you taken your pill?’”

### 3.4. Challenges during Participation

Participants discussed four main challenges they experienced as a result of their involvement with the TDF2 trial: 1) side effects, 2) adherence to the study medication, 3) lengthy study visits, and 4) others’ worry and disbelief about participants’ health.

Nearly half of the participants described experiencing side effects such as gas, nausea, increased hunger, headaches, reduced sex drive, or weakness in the joints as a challenge to participation. Those who experienced side effects said they reported symptoms to the study staff and then waited for the side effects to subside. A male from Gaborone described a commonly shared perspective, “The problem that I had with taking the pill was a running stomach [diarrhea]. It took about seven to eight days [to stop] and when I asked [the study staff] they told me that it was likely to happen… and that it would stop. And indeed it did stop.” Francistown participants noted these symptoms were not worrisome because they were expected. One female explained, “They had told us that when you are starting [the pills], they may cause diarrhea and this and this and I just told myself that what I was experiencing was one of those signs.”

Participants, in particular males and those who self-reported non-adherence, said that they had difficulties correctly taking their daily pill. Participants attributed non-adherence to disruptions in one’s usual schedule (e.g., leaving pills at home during occasional travel). In spite of recognizing challenges with correctly taking the study pill, participants downplayed these issues, noting that disruptions were infrequent and short-lived. A non-adherent female from Francistown summarized, “I did not have any problem [taking the pills]… I used to forget, maybe a day, [but] I did not take it to be a major problem because it did not happen continuously.”

The clinic management and lengthy study visits were also identified as challenges. Participants noted that the order in which they were seen by the nurses and counselors did not always correspond to their arrival time, creating the perception that some participants were unfairly moving more quickly through the study visit process. A woman from Gaborone explained, “There is this system… when you are in the nurses’ line, the counselor would take you… Some of the counselors would promise to return you to where you were [in line]. Some of the interviewers did not return you to where you were. You would then start the line from behind. I was in a hurry once and I was put far behind. Some of the people who were behind me were put in front of me.” Men in particular talked about how the lengthy study visits, or visits that took longer than anticipated, created difficulties with their employers. One male from Francistown shared, “Sometimes it was too crowded [at the clinic], such that it was a problem for me at work. I could be away for up to four hours or even five hours there. It affected my work and [my bosses] were starting to complain, but sometimes I would avoid it by just taking my own leave to go to BOTUSA [study clinic].”

A final challenge discussed by participants was that friends and family questioned their eligibility to take part in an HIV trial or worried that the study pills would cause them harm. Participants described the commonly held belief that healthy people should not take medications. This led some friends and family to question how taking daily medication would affect the participants’ health. A male from Gaborone explained, “The concern that I had in my life was with my parents. My parents completely did not want [me to participate] and my friends as well. They asked me why I would take a pill when I was not sick, and did I not realize that this pill could kill me?” Others wondered if participants were enrolling in the study to receive treatment because they were actually HIV-positive. Another male from Gaborone shared, “Others were critical; they asked me why I took the pill when [there] was nothing wrong with me? They concluded that I must have some disease.”

## 4. Discussion

### 4.1. Study Summary

Our analysis of 53 in-depth interviews with a subset of TDF2 trial participants sought to examine participants’ knowledge, attitudes, and experiences of the trial, identify facilitators and barriers to enrollment and retention, and compare participant responses by study site, sex, and study drug adherence. More than 60% of our qualitative sample had been enrolled in the trial for 13 months at the time they were interviewed. During one or more monthly study visits, 68% reported missing one or more doses to the study drug. Qualitative findings show that despite perceptions of being well informed on the aims of the trial and the enrollment eligibility criteria, participants lacked an in-depth understanding about the trial prior to their initial clinic visit. In addition to misinformation by participants and others, negative views by the community toward research and researchers, in particular concerns about the researchers’ agenda (HIV infection intention rather than HIV prevention) were commonly cited. Findings further suggest that concerns about potential short term and long study product side effect symptoms had implications for both trial enrollment and study product adherence. Participants who reported that they had not missed any doses of the study product were less likely than those who reported to missing one or more doses to express concerns about potential side effects. Male participants and those who self-reported non-adherence indicated that they had difficulties correctly taking their daily pill when their usual schedule was disrupted (e.g., leaving pills at home during occasional travel). However, in spite of recognizing challenges with correctly taking the study pill, such issues were downplayed given their infrequent and short-lived occurrence. Participants expressed that Clinic management and lengthy study visits were also identified as participation challenges. Overall, participants characterized their trial participation favorably. Personal commitment or an internal decision to see it through were seen as motivating continued participation.

### 4.2. Comparison with Literature

Studies investigating retention and adherence to PrEP interventions have uncovered that multi-faceted interventions are most effective [19] [20]. Evaluations of adherence to anti-retroviral therapy interventions suggest that ensuring an accurate understanding of PrEP benefits and adherence requirements, provider monitoring of adherence, social support, and a routine pill-taking schedule should be included in PrEP adherence efforts [8]. Our findings point to several considerations for optimizing recruitment, retention, and adherence in PrEP trials and programs, including pre-enrollment education to minimize misperceptions about study design and purpose (*i.e*., trial myth reduction), providing accurate estimates of participant obligations and side effect symptoms, ensuring participant understanding of the effects of non-adherence, gauging personal commitment and interest in study outcomes, and developing a strong external social support network for participants. In addition, our findings emphasize the importance of good rapport with the clinical staff and addressing clinic management issues to ensure that lengthy visits and participant wait times are appropriately addressed.

Participants identified several challenges to enrollment, retention, and adherence in PrEP efficacy trials. Our participants were frustrated by longer than anticipated study visits, which caused scheduling conflicts. Many noted that they did not have accurate information prior to their first study clinic visit. Other PrEP trials [10] [11] have found that knowledge of the study product and trial objectives positively affected drug adherence. Our findings also highlight the importance of providing realistic information about trial logistics and participant burden. Participants also noted that negative beliefs about the study existed in their community. A study of microbicide gels in Africa found that rumors about the trial caused participants to miss study visits [13]. It is important to consider that even though participants decide to enroll in a clinical trial, information they initially receive about the study from community members may leave lingering impressions or doubts about their decision. Providing accurate, realistic, and thorough information prior to the first interaction at the study clinic will not only allow potential participants more time to consider whether enrollment is right for them, it may also mitigate problems with retention caused by rumors. In addition, implementing community engagement activities prior to the start of the trial will permit researchers to identify and address any concerns in the community that could impact enrollment and retention.

Participants discussed the challenge of adherence. In trial questionnaires, participants provided reasons for non-adherence, including forgetting to take the pills, encountering logistical challenges (e.g., traveling or spending a night away from home, problems with refilling prescription) or side effect experiences or concerns [18]. In the in-depth interviews, however, they emphasized that non-adherence was an exception rather than the norm in their pill-taking behavior. Over-reporting of adherence to HIV treatment medication is common [21]. These findings suggest that non-adherence and over-report of adherence may be related to participants’ misunderstanding of the definition and consequences of non-adherence. Given importance of adherence for PrEP efficacy, it is imperative that researchers and participants as well as health care providers and patients have a shared understanding of the definition of PrEP adherence. Uncertainty about the safety of the study product may also influence participants’ adherence and retention [22]. In our study, non-adherent participants were less likely than adherent participants to discuss concerns about side effects but were more likely to discuss their concerns about being infected with HIV or other diseases as a result of trial participation. In a multivariable analysis of the main study data, greater adherence (measured by pill count but not self-report) was found to be significantly associated with a lack of concern about side effects and not experiencing nausea, dizziness, and vomiting, the three most commonly reported adverse events [18].

In spite of these challenges, participants characterized their involvement with the trial as a positive experience and identified several facilitators to enrollment and retention. As in other PrEP studies [6] [11] [23] [24], access to medical services and altruism were found to motivate participants to enroll and remain in the TDF2 trial. Participants in this study also said that personal commitment to follow through and interest in the study results were motivators. Further investigations into how participants’ perceptions of personal commitment and interest in findings at enrollment are related to retention may be useful to identify those who will need to receive targeted retention strategies throughout the course of a trial.

We found that social support (both encouragement and discouragement) had a lasting influence on participants’ experiences and their perceptions regarding those experiences. Other studies have shown that social support is an important factor in for adherence to medication and retention in HIV Prevention clinical trials [6] [11] [13] [25]. In this study, adherent participants expressed that support from others helped promote the risk-reduction practices emphasized in the counseling sessions, including condom use, decrease in number of sexual partners, and remembering to take the study pills. Likewise, discouragement from others caused participants question their decision to enroll or remain in the trial. Discouragement often stemmed from others’ misunderstanding of the study purpose. This highlights the importance of including participants’ support networks in retention and adherence strategies, including ensuring that participants can convey straightforward, factual information about the study to clarify misconceptions and garner support.

### 4.3. Study Limitations

This study has several limitations. First, our findings are not generalizable to other populations. However, we had similar findings to other studies examining barriers and facilitators to adherence and retention in HIV prevention clinical trials [6] [10] [11] [13] [23]-[25]. Second, interviews were conducted with trial participants who were retained in the study. These men and women may have had a more positive perceptions of and experiences with the TDF2 trial than those who were not retained or did not agree to participate in an interview. Although this study focused on the perspectives and experiences of participants in a Phase 3 PrEP clinical trial, our findings highlight several factors to consider for retention and adherence in future PrEP trials and programs, including pre-enrollment community and participant engagement and education that clarifies information about the study, gauging a participant’s personal commitment and interest in study outcomes, and developing good rapport with participants as well as an external social support network. Third, this manuscript is one of several analyses undertaken for this study and the three year lapse between sub-study data collection and publication of findings is worthy of mention. A social construction orientation of qualitative research acknowledges that social interaction as well as changes in access to and content of information may influences how people recall and make sense of their experiences. Today, participants in our sub-study may emphasize different knowledge, attitudes, and experiences about the trial now that they know the PrEP efficacy outcomes for this trial as well as other PrEP clinical trials. No longer being active study participants may likewise influence their views and recall. Finally, all main study enrollees underwent rigorous medical screening and were determined not to have health compromising medical conditions. Co-management of HIV with and other diseases, such as tuberculosis malaria, and hepatitis, is common in clinical practice; however, addressing how treatment for such diseases may affect HIV-uninfected persons on PrEP goes beyond the scope of our study.

### 4.4. Conclusion

Understanding participants’ experiences with participation in a PrEP efficacy trial will inform strategies to increase adherence and retention in future clinical trials and the implementation of PrEP programs. Multi-faceted efforts that include education, information, and support may assist in translating promising clinical trial results into public health impact.

## Figures and Tables

**Table 1:** In-depth interview guide questions selected for qualitative data analysis, Botswana TDF2 qualitative sub-study, April 2010.

Domain of inquiry	Questions
Pre-enrollment attitudes and knowledge	How did you hear about the study?
	What did you hear about the study?
	What are some of the beliefs or stories (myths/conspiracies) about why this study is done in Botswana?
Participation and retention facilitators and barriers	What motivated you to join this study?
	What did you think the benefits of joining the study would be?
	What concerns did you have about joining the study?
	What challenges did you face while in the study?
	What are the difficulties you have faced in taking your pills?
	How have you managed to keep up with participation in the study?
Others’ reactions to study participation	Who have you told about your participation in the study?
	How have others reacted to your participation in the study?

**Table 2:** Demographic characteristics of qualitative interview participants, Botswana TDF2 qualitative sub-study, April 2010.

Characteristic	Total N = 53	Francistown N = 26	Gaborone N = 27

	n (%)	n (%)	n (%)
Gender			
Male	24 (45)	11 (42)	13 (48)
Female	29 (55)	15 (58)	14 (52)
Age (years) at time of interview			
21 – 29	41 (77)	19 (73)	22 (81)
30 – 39	11 (21)	7 (27)	4 (15)
40+	1 (2)	0 (0)	1 (4)
Education			
Primary or less	3 (6)	2 (8)	1 (4)
Secondary	42 (79)	21 (81)	21 (78)
Post secondary	8 (15)	3 (12)	5 (19)
Length of time (months) in study			
6 – 12	17 (32)	8 (31)	9 (33)
13 – 18	11 (21)	5 (19)	6 (22)
19 – 24	15 (28)	8 (31)	7 (26)
25+	10 (19)	5 (19)	5 (19)
Reported non-adherence in main study			
Yes	36 (68)	16 (62)	20 (74)
No	17 (32)	10 (38)	7 (26)
